# The association of maternal-fetal attachment with smoking and smoking cessation during pregnancy in The FinnBrain Birth Cohort Study

**DOI:** 10.1186/s12884-020-03393-x

**Published:** 2020-11-30

**Authors:** Heidi Jussila, Juho Pelto, Riikka Korja, Eeva Ekholm, Marjukka Pajulo, Linnea Karlsson, Hasse Karlsson

**Affiliations:** 1 Doctoral Programme of Clinical Investigation, Department of Child Psychiatry, University of Turku, Turku, Finland; 2 FinnBrain Birth Cohort Study, Turku Brain and Mind Center, Department of Clinical Medicine, University of Turku, Lemminkäisenkatu 3, 20014 Turku, Finland; 3 Department of Psychology , University of Turku , Turku, Finland; 4 Department of Obstetrics and Gynecology , Turku University Hospital , Turku, Finland; 5 Department of Child Psychiatry , University of Turku , Turku, Finland; 6 Centre for Population Health Research , University of Turku and Turku University Hospital , Turku, Finland; 7 Department of Child Psychiatry , Turku University Hospital and University of Turku , Turku, Finland; 8 Department of Psychiatry , Turku University Hospital and University of Turku , Turku, Finland

**Keywords:** Smoking in pregnancy, Smoking cessation, Smoking, Pregnancy, Prenatal, Maternal-fetal attachment, Prenatal parenting, Prenatal stress, Prenatal psychological distress, Education

## Abstract

**Background:**

Smoking in pregnancy constitutes a preventable risk factor for fetal/child development and maternal-fetal attachment (MFA) seems to contain a momentum that can break the chain of adverse outcomes by promoting maternal prenatal health practices. This study aimed to explore the association of MFA with smoking at any time during pregnancy and smoking cessation in early pregnancy, and the modifying role of MFA on the expected effects of education and prenatal psychological distress (PPD) on prenatal smoking behavior.

**Methods:**

The pregnant women (*n* = 3766) participated in the The FinnBrain Birth Cohort Study in Finland between December 2011 and April 2015. The binary outcomes, smoking at any time during pregnancy and smoking cessation in early pregnancy, were obtained from self-reports at gestational weeks (gwks) 14 and 34 and The Finnish Medical Birth Register. MFA was assessed with the Maternal-Fetal Attachment Scale (MFAS) at gwks 24 and 34. Logistic regression analyses were used to determine the association between MFA and maternal prenatal smoking behavior.

**Findings:**

The prevalence of smoking was 16.5%, and 58.1% of the smokers quit smoking during pregnancy. The independent associations of total MFA scores with prenatal smoking behavior were not established (aOR = 1.00-1.02, multiplicity adjusted *p* > 0.05). A higher score in the altruistic subscale of MFA, *Giving of self*, associated with a higher probability of smoking cessation (24 gwks: aOR = 1.13, 95% CI [1.04, 1.24], *p* = 0.007, multiplicity adjusted *p* = 0.062; 34 gwks: aOR = 1.17, 95% CI [1.07, 1.29], *p* < 0.001, multiplicity adjusted *p* = 0.008). The modifying effect of MFA on the observed associations between PPD and smoking in pregnancy and between maternal education and smoking in pregnancy / smoking cessation in early pregnancy was not demonstrated.

**Conclusions:**

The altruistic dimension of maternal-fetal attachment associates with an increased probability of smoking cessation during pregnancy and therefore strengthening altruistic maternal-fetal attachment may constitute a promising novel approach for interventions aiming at promoting smoking cessation during pregnancy.

## Background

Fetal exposure to maternal tobacco smoking is one of the key risk factors for neonatal health [1–3] and impairs health and achievements in childhood and later in the course of life [2, 3]. Globally, the prevalence of smoking in pregnancy is highest in the European and American regions, around 6–8% [4], and affects nearly 15% of unborn children in Finland [5]. Inequality in health and attainment originates early since the exposure to maternal smoking during pregnancy accumulates in more vulnerable populations in our society [1, 5–7]. The association between educational disadvantage and maternal prenatal smoking has been widely recognized [1, 7, 8]. In Finland, low maternal education constitutes the strongest predictor of smoking in pregnancy [9]. While educational attainment is a well-established and robust predictor of persistent smoking during pregnancy, underlying mechanisms are not well-elucidated [7]. However, extensive literature has shown that lower educational attainment constitutes a powerful marker of psychosocial and familial risk [7]. For example, educational attainment is closely related to heritable personality traits related to self-control [10]. Thus, low educational attainment may be a marker for both familial risk [11] and difficulty adapting behavior to environmental contingencies. This possibility is supported by one prior study linking lower self-directedness to persistent smoking during pregnancy [12]. Further, several previous studies have revealed a deleterious association between prenatal psychological distress (PPD) and smoking habits during pregnancy [6, 13–16]. Unfortunately, a tendency towards co-occurrence of prenatal smoking, stress and socioeconomic disadvantage have been found to indicate an accumulative burden for maternal and child health [1, 17].

Prenatal parenting, which hereby refers to the expectant mother’s attachment towards her unborn child, has been considered a counterforce for prenatal adversities and protective for child development, especially in populations at risk [18]. Stronger maternal-fetal attachment (MFA), defined by Cranley (1981) as “the extent to which women engage in behaviors that represent an affiliation and interaction with their unborn child”, is associated with favorable maternal prenatal health behavior [19, 20], neonatal [19] and child developmental outcomes [21]. In a recent meta-analysis, maternal-fetal attachment has been reported to be the strongest predictor for positive maternal prenatal health practices with a large effect [20]. Previous studies have shown that pregnant women experiencing stronger attachment towards the fetus are less likely to smoke during pregnancy [19, 22]. In addition, stronger MFA has associated with lower salivary cotinine levels and a tendency to smoke a lower maximum number of cigarettes per day [23]. Pregnant smoking women considering smoking cessation have been found to experience stronger attachment towards the fetus than pregnant smokers in the pre-contemplation stage [24]. The stronger altruistic dimension of MFA especially, that is “*Giving of self”*, has predicted a higher probability of smoking cessation among pregnant smokers [25]. This specific aspect of MFA reflects the mother’s feeling of the pregnancy being worth all the effort, an acceptance of body changes and image during pregnancy, a willingness and commitment changing habits, following a healthy diet, and giving up unhealthy behaviors, essentially for the sake of the unborn child [26]. It is noteworthy that more profound prenatal attachment may also protect the woman from depressive symptoms in late pregnancy [27], which, in turn, may contribute to abstinence from tobacco smoking [16, 28]. In addition, MFA may have potential to modify mechanisms underlying the adverse association between low maternal educational attainment and smoking behavior in pregnancy. It is of significance that MFA, in contrast to maternal educational attainment, can potentially be affected through prenatal interventions [29].

Importantly, smoking in pregnancy constitutes a preventable risk factor in the intergenerational transmission of health related inequality [1–3] and MFA seems to contain a momentum that can break the chain of adverse outcomes by promoting maternal prenatal health practices [19, 20, 23, 25] and fetal/child developmental trajectories [19, 21]. Nevertheless, research to date has been based on a few studies with relatively small samples, and the significance of MFA in the determination of prenatal smoking behavior and the potential implication for smoking cessation interventions has not yet been extensively studied. In addition, MFA constitutes a promising candidate target for prenatal interventions for other reasons also. Tobacco use disorder in pregnancy has been found to associate with impaired postnatal parenting, such as an increased risk for a harsh parenting style and child maltreatment [30]. It is of note that a stronger MFA has been found to predict better parent-child interaction quality [31] and postpartum maternal sensitivity [32] which predicts child attachment security [33]. Thus, intervening to increase MFA during pregnancy has the potential to address both direct teratologic effects of prenatal tobacco exposure and impaired maternal parenting behaviors that may amplify childhood adversity of children exposed to maternal prenatal tobacco smoking.

The first aim of the current study was to investigate the association of MFA with maternal tobacco smoking at any time during pregnancy and smoking cessation in early pregnancy. We hypothesized that a stronger MFA associates with a lower risk of smoking in pregnancy and an increase in smoking cessation during pregnancy. Second, we presumed that a stronger MFA protects and promotes maternal and child health by beneficially modifying the effects of PPD and maternal education on prenatal smoking behavior, especially in the presence of maternal educational disadvantage and mental health related adversity. Therefore, we explored the modifying effect of MFA on the associations between maternal education/PPD and smoking/cessation of smoking during pregnancy. The hypotheses were pre-defined in the Open Science Framework portal (https://osf.io/v4nd3/).

## Methods

### Research design

#### The FinnBrain Birth Cohort Study

The FinnBrain Birth Cohort was collected in the Southwest Finland and on Åland Island in Finland between December 2011 and April 2015 (www.finnbrain.fi). Pregnant women were screened for eligibility at a screening ultrasound at the 12th gestational week (gwk). A normal ultrasound screening result and the ability to respond to the study questionnaires in Finnish or Swedish were the criteria for eligibility. The present study was cross-sectional, and linked the survey data with the data obtained from The Finnish Medical Birth Register maintained by The National Institute of Health and Welfare in Finland. A written informed consent was obtained from all participants. The Ethics Committee of the Hospital District of Southwest Finland approved the The FinnBrain Cohort Study.

### Participants

During the collection of the birth cohort sample, 8895 pregnant women attended the ultrasound screening visits in the geographical area, and 65% (5790 of 8895) of them were informed about The FinnBrain Cohort Study. Among the eligible women (*n* = 5790), 3808 (65.8%) agreed to participate. Only the women with a singleton pregnancy were included in the present study, and the total number of pregnant women surveyed was 3766.

By their delivery date, 7.6% (288 of 3766) of the participants had chosen to discontinue. The discontinuation rate did not differ between the non-smokers and the smokers (6.9% vs. 5.4%, *F*[1] = 1.8, *p* = 0.179, respectively), or between those who had quit smoking and the persistent smokers (6.5% vs. 3.1%, *F*[1] = 3.5, *p* = 0.063, respectively). Those participants and the pregnant smokers who had completed the questionnaire measure of MFA at least once during pregnancy were included in the imputation and regression models (24 gwks: *n* = 2858 [76%] and 34 gwks: *n* = 2836 [75%]; 24 gwks: *n* = 429 [70%] and 34 gwks: *n* = 426 [70%], respectively).

### Measures

#### Dependent variables

##### Smoking at any time during pregnancy and smoking cessation in early pregnancy

The data on smoking at any time during pregnancy was derived by combining the survey self-reports and the records in The Finnish Medical Birth Register. The pattern of tobacco smoking was surveyed twice during pregnancy. At 14 gwks, the women responded to whether they had smoked during the index pregnancy. At 34 gwks, the participants reported whether they had been smoking during the last 20 weeks. The Finnish Medical Birth Register contained data on smoking in pregnancy in the format: no prenatal smoking, smoking cessation in the first trimester (≤ 12 + 0 gwks), continuation of smoking after first trimester (≥ 12 + 1 gwks) and no data available. We created a binary outcome variable *Smoking at any time during pregnancy* which was affirmative when the participant had self-reported or had a register record of smoking during the index pregnancy. Second, we constructed a binary outcome variable *Smoking cessation in early pregnancy*. The pregnant smoker was categorized as a persistent smoker if there was a register record on a continuation of smoking after the first trimester (≥ 12 + 1 gwks) or an affirmative survey self-report on smoking in pregnancy at 34 gwks.

#### Independent variables

##### Maternal-fetal attachment

MFA was assessed with the Maternal-Fetal Attachment Scale (MFAS), which is a 24-item self-report questionnaire [26]. The items are scored on a five-point scale (1–5) with a total score ranging from 24 to 120, a higher sum score indicating a stronger attachment towards the fetus. MFAS contains five subscales: “*Role taking*”, “*Differentiation of self from fetus*”, “*Interacting with the fetus*”, “*Attributing characteristics to the fetus*” and “*Giving of self*” [26]. The MFAS questionnaire was administered to the participants at the 24th and 34th gwks. The MFAS scores were included in the statistical models as continuous variables. Reliability of the MFAS was found to be good (Cronbach’s α = 0.85). For the MFAS subscales, internal consistencies varied between poor and good (“*Role taking”*, Cronbach’s α = 0.81–0.83; “*Differentiation of self from fetus”*, Cronbach’s α = 0.50–0.53; “*Interacting with the fetus”*, Cronbach’s α = 0.55; “*Attributing characteristics to the fetus”*, Cronbach’s α = 0.64–0.66; and “*Giving of self”*, Cronbach’s α = 0.51–0.52).

##### Additional independent variables

Maternal age, education, single parenthood, parity and concurrent symptoms of depression and anxiety were identified from previous literature and adjusted to address any potential confounding [5, 7–9, 15, 16, 28, 34]. The information concerning maternal education was collected with a self-report questionnaire at the 14th gwk, and classified into: low [high school/vocational, < 12 years], middle [polytechnics], and high [university]. The binary variable describing single parenthood was affirmative in cases where the participant did not have an intimate relationship based on the survey self-report or she was not cohabiting based on the register data. Parity (primiparous or multiparous) and maternal age were derived from The Medical Birth Register. Symptoms of PPD were assessed with the Edinburgh Pre/Postnatal Depression Scale (EPDS) [35] and with the anxiety subscale of the Symptom Checklist-90 (SCL-90) [36, 37] at the 24th and 34th gwks. The symptoms of PPD were included in the statistical models as continuous variables. The EPDS cut-off score ≥ 10 was used to describe the sample in terms of prevalence of prenatal depression [38]. The internal consistencies for the EPDS (Cronbach’s α = 0.84) and for the anxiety section of SCL-90 (Cronbach’s α = 0.84–0.85) were found to be relatively good.

### Analyses

In the background variables, the comparisons between the groups with nominal variables were performed using a Pearson’s chi-square test and for normally distributed continuous variables using a two-tailed independent sample’s t-test. A Mann-Whitney U test was used to analyze the group differences in MFA and PPD. A one-way ANOVA was performed to investigate the level of MFA by the level of maternal education (low, middle, high).

Multiple imputation (30 imputed datasets) was used to treat the missing values in the predictor variables. The MFAS scores were imputed by the factors (subscales), and the total sum scores were calculated based on the imputed factors. Separate imputation models were constructed for smoking and smoking cessation during pregnancy. All the variables included in the analysis models were also included in the imputation models.

Next, logistic regression models were used to estimate the association of MFA (both the total and the subscale scores of MFAS at the 24th and 34th gwks) with the binary dependent variables, smoking at any time during pregnancy and smoking cessation in early pregnancy, while controlling for maternal age, educational status, single parenthood, parity and concurrent depressive and anxiety symptoms. Finally, to explore whether MFA modifies the associations between maternal education/PPD and smoking behavior in pregnancy, we added *MFAS* × Education and *MFAS* × PPD interactions, respectively, in the models. Descriptive statistical analyses were conducted using an SPSS version 24, and the multiple imputation and the statistical modeling were performed with R 3.5.2. P-values < 0.05 were considered statistically significant. For the main analyses, the correction for multiplicity was performed separately for each of the four hypotheses. Correlations between the MFAS sum and subscale scores at both trimesters (*r* = 0.28 – 0.83) were taken into account in the corrections by calculating the effective number of tests [39] and using that as the correction factor for the p-values. The correction factor thus reduced from 12 to 8.84 in the main effect analyses (hypotheses 1 and 2) and from 8 to 5.24 in the interaction analyses (hypotheses 3 and 4).

## Results

### Descriptive sample characteristics

The prevalence of smoking in pregnancy was 16.5% (612 of 3698). Among the women who were smoking at the time of conception, 58.1% (354 of 609) quit smoking during pregnancy, while 41.9% (255 of 609) continued smoking beyond 12 − 14 gwks. The data concerning smoking in pregnancy was missing for 1.8% (68 of 3766) of the participants, and regarding smoking cessation for 0.5% (3 of 612) of the pregnant smokers. The smoking women were younger than the non-smokers, *M*_*years*_ = 28.2(*SD* 5.2) vs. *M*_*years*_ = 30.7(*SD* 4.5) respectively, *t*(799.1) = 11.1, *p* = 0.000. A difference in age was not displayed between those women who quit smoking in early pregnancy compared to the women who continued smoking in late pregnancy *M*_*years*_ = 28.4(*SD* 5.0) vs. *M*_*years*_ = 27.8(*SD* 5.5) respectively, *t*(511.6) = 1.6, *p* = 0.12. The socio-demographic characteristics of the sample are presented in Table 1. The descriptive statistics regarding MFA and PPD are reported in Tables 2 and 3. The group means of maternal-fetal attachment differed significantly by the level of maternal education (low, middle, high) (MFAS 24 gwks: *F*(2, 2603) = 10.6, *p* < 0.001; 34 gwks: *F(*2, 2444) = 4.0, *p* = 0.019. Post hoc comparisons demonstrated the statistically significant difference in the MFAS total score between the low level and the high level of maternal education (*M* = 87.7, *SD* = 10.2 vs. *M* = 85.6, *SD* = 10.3, *p* < 0.001, respectively) and between the middle level and the high level of education (*M* = 87.5, *SD* = 10.9 vs. *M* = 85.6, *SD* = 10.3, *p* = 0.001, respectively) in the second trimester. In addition, differences were observed in the level of MFA between the low and the high level of maternal education (*M* = 92.5, *SD* = 10.2 vs. *M* = 91.4, *SD* = 10.3, *p* = 0.046, respectively) and between the middle and the high level of maternal education (*M* = 92.5, *SD* = 10.3 vs. *M* = 91.4, *SD* = 10.3, *p* = 0.047, respectively) in the third trimester.

**Table 1:** Descriptive characteristics of the pregnant non-smokers and smokers, and those who quit smoking and those who continued

	Non-smokers	Smokers	Between-groups difference		Quit smoking	Continued smoking	Between-groups difference	
	*N = *3086	*N = *612			*N *= 354	*N *= 255		
	*n/N (%)*	*n/N (%)*	*df*	*χ*^*2*^*p*	*n/N (%)*	*n/N (%)*	*df*	*χ*^*2*^ *p*
Primiparous	1432/3086 (46.4%)	353/612 (57.7%)	1	<0.001**	231/354 (65.3%)	112/255 (47.1%)	1	<0.001**
Single parent	80/3080 (2.6%)	68/605 (11.2%)	1	<0.001**	17/353 (4.8%)	51/249 (20.5%)	1	<0.001**
Education			2	<0.001**			2	<0.001**
Low	828/2582 (32.1%)	325/462 (70.3%)			190/298 (63.8%)	133/161 (82.6%)		
Middle	803/2582 (31.1%)	81/462 (17.5%)			62/298 (20.8%)	19/161 (11.8%)		
High	951/2582 (36.8%)	56/462 (12.1%)			46/298 (15.4%)	9/161 (5.6%)		
Income			3	<0.001**			3	0.015*
<1000€	544/2577 (21.1%)	133/460 (28.9%)			72/297 (24.2%)	59/160 (36.9%)		
1001-1500€	409/2577 (15.9%)	112/460 (24.3%)			71/297 (23.9%)	41/160 (25.9%)		
1501-2000€	870/2577 (33.8%)	140/460 (30.4%)			101/297 (34.0%)	39/160 (24.4%)		
>2000€	754/2577 (29.3%)	75/460 (16.3%)			53/297 (17.8%)	21/160 (13.1%)		
Self-reported prior psychiatric disorder (any)	419/2580 (16.2%)	126/460 (27.4%)	1	<0.001**	74/297 (24.9%)	50/160 (31.3%)	1	0.146
Self-reported prior depression	291/2580 (11.3%)	93/460 (20.2%)	1	<0.001**	51/297 (17.2%)	40/160 (25.0%)	1	0.046*
Self-reported prior anxiety disorder	137/2580 (5.3%)	38/460 (8.3%)	1	0.012*	23/297 (7.7%)	14/160 (8.8%)	1	0.707

The descriptive sample statistics are based on the original data, not the imputed dataset

*N* = information available or relevant total *N*, *n* = a number of pregnant women with an affirmative response

* *p*-value <.05, **p-value <.01

**Table 2:** The descriptives statistics of MFA and PPD, and the comparisons between the pregnant smokers and the non-smokers

							Between-groups difference	
	*N *= 3086			*N *= 612				
	*n*	*Mean (SD)*	*Median (range)*	*n*	*Mean (SD)*	*Median (range)*	*p*^*a*^	*r*
**24 gwks**								
MFAS	2331	86.9 (10.6)	87 (51-116)	409	88.0 (10.0)	88 (44-113)	0.074	0.03
*Role taking*	2336	16.7 (2.8)	17 (6-20)	410	16.8 (2.7)	17 (7-20)	0.703	0.01
*Differentiation*	2340	15.4 (2.5)	15 (6-20)	410	15.7 (2.4)	16 (7-20)	0.032*	0.04
*Interacting*	2338	16.6 (3.1)	17 (5-25)	410	17.1 (3.0)	17 (8-24)	0.004**	0.05
*Attributing*	2335	18.5 (3.4)	19 (6-30)	409	19.0 (3.2)	19 (11-29)	0.006**	0.05
*Giving*	2338	19.7 (2.5)	20 (9-25)	410	19.5 (2.5)	20 (8-25)	0.091	0.03
EPDS	2317	4.8 (4.1)	4 (0-25)	408	6.0 (4.3)	5 (0-23)	<0.001**	0.11
SCL-90 Anxiety	2314	3.8 (4.2)	3 (0-30)	408	4.7 (4.6)	4 (0-24)	<0.001**	0.08
**34 gwks**								
MFAS	2188	92.0 (10.2)	93 (52-116)	373	93.1 (10.2)	93 (55-117)	0.137	0.03
*Role taking*	2202	17.1 (2.6)	18 (7-20)	374	17.1 (2.5)	18 (8-20)	0.886	0.00
*Differentiation*	2204	15.9 (2.4)	16 (8-20)	374	16.2 (2.2)	16 (10-20)	0.111	0.03
*Interacting*	2202	18.1 (3.1)	18 (5-25)	374	18.5 (2.8)	19 (10-25)	0.037*	0.04
*Attributing*	2198	21.1 (3.4)	22 (9-30)	373	21.8 (3.4)	22 (12-30)	0.001**	0.07
*Giving*	2198	19.8 (2.4)	20 (10-25)	374	19.6 (2.5)	20 (10-25)	0.171	0.03
EPDS	2191	4.7 (4.0)	4 (0-26)	373	5.9 (4.5)	5 (0-23)	<0.001**	0.10
SCL-90 Anxiety	2186	3.1 (3.8)	2 (0-27)	374	3.8 (4.6)	3 (0-33)	0.012*	0.05
		**n/N**	**%**		**n/N**	**%**	***χ2*** ***p***	**Cramer’s ϕ**
**24 gwks **EPDS≥10		299/2317	12.9%		73/408	17.9%	0.007**	0.05
**34 gwks **EPDS≥10		287/2191	13.1%		84/373	22.5%	<0.001**	0.09

The estimates are based on the original data, not the imputed dataset

Attrition in the MFAS questionnaire responses was higher among the smoking women in comparison with the non-smokers (24 gwks: 33.2% vs. 24.5%, χ2 = 20.2,* p* < 0.001; 34 gwks: 39.1% vs. 29.1%, χ2 = 23.8, *p* < 0.001, respectively)

*MFAS* Maternal-Fetal Attachment Scale and the subscales of Maternal-Fetal Attachment Scale: *Role taking*, *Differentiation* Differentiation of self from fetus, *Interacting* Interacting with the fetus, *Attributing* Attributing characteristics to the fetus and *Giving* Giving of self, *EPDS* Edinburgh (Pre) Postnatal Depression Scale, *SCL-90 Anxiety* Symptom Checklist -90, the Anxiety Subscale

* *p*-value <.05, **p-value <.01

^a^ Mann-Whitney U test

**Table 3:** The descriptives statistics of MFA and PPD, and the comparisons between the smokers who quit and the persistent smokers

							Between-groups difference	
	*N *= 354			*N *= 255				
	*n*	*Mean(SD)*	*Median(range)*	*n*	*Mean(SD)*	*Median(range)*	*p*^*a*^	*r*
**24 gwks**								
MFAS	257	88.6 (10.0)	88 (57-113)	152	87.0 (10.0)	87 (44-112)	0.102	0.08
*Role taking*	258	16.6 (2.7)	17 (8-20)	152	16.7 (2.6)	17 (7-20)	0.237	0.06
*Differentiation*	258	15.7 (2.4)	16 (9-20)	152	15.6 (2.3)	16 (7-20)	0.644	0.02
*Interacting*	258	17.1 (3.1)	17 (8-24)	152	17.0 (2.9)	17 (9-24)	0.574	0.03
*Attributing*	257	19.1 (3.2)	19 (11-29)	152	19.0 (3.2)	19.0 (11-29)	0.773	0.01
*Giving*	258	19.8 (2.4)	20 (13-25)	152	18.8 (2.6)	19 (8-25)	<0.001**	0.18
EPDS	256	5.6 (4.1)	5 (0-20)	152	6.8 (4.5)	6 (0-23)	0.006**	0.14
SCL-90 Anxiety	256	4.3 (4.5)	3 (0-24)	152	5.4 (4.7)	4 (0-20)	0.007**	0.13
**34 gwks**								
MFAS	236	93.9 (9.7)	93 (67-115)	137	91.8 (10.9)	91 (55-117)	0.093	0.09
*Role taking*	236	17.3 (2.5)	18 (8-20)	138	17.0 (2.5)	17 (8-20)	0.136	0.08
*Differentiation*	236	16.2 (2.3)	16 (10-20)	138	16.1 (2.2)	16 (10-20)	0.883	0.01
*Interacting*	236	18.5 (2.7)	19 (12-25)	138	18.4 (3.0)	18 (10-25)	0.555	0.03
*Attributing*	236	21.9 (3.3)	22 (12-28)	137	21.6 (3.7)	22 (12-30)	0.542	0.03
*Giving*	236	20.1 (2.5)	20 (12-25)	138	18.8 (2.5)	18 (10-25)	<0.001**	0.26
EPDS	235	5.4 (4.2)	5 (0-20)	138	6.8 (4.9)	6 (0-23)	0.007**	0.14
SCL-90 Anxiety	235	3.5 (4.5)	2 (0-33)	139	4.5 (4.7)	3 (0-26)	0.016*	0.12
		**n/N**	**%**		**n/N**	**%**	***χ2p***	**Cramer’s **ϕ
**24 gwks **EPDS≥10		36/256	14.1%		37/152	24.3%	0.009**	0.13
**34 gwks **EPDS≥10		42/235	17.9%		42/138	30.4%	0.005**	0.15

The estimates are based on the original data, not the imputed dataset

Attrition in the MFAS questionnaire responses was higher among the persistent smokers in comparison with the women who quit smoking during pregnancy (24gwks: 40.9% vs. 26.0, χ2 = 14.5, *p* < 0.001; 34 gwks: 46.8% vs. 29.6%, χ2 = 18.2, *p* < 0.001, respectively)

*MFAS* Maternal-Fetal Attachment Scale and the subscales of Maternal-Fetal Attachment Scale: *Role taking*, *Differentiation* Differentiation of self from fetus, *Interacting* Interacting with the fetus, *Attributing* Attributing characteristics to the fetus and *Giving* Giving of self, *EPDS* Edinburgh (Pre) Postnatal Depression Scale, *SCL-90 Anxiety* Symptom Checklist -90, the Anxiety Subscale

* *p*-value <.05, **p-value <.01

^a^ Mann-Whitney U test

### The association of maternal-fetal attachment with smoking at any time during pregnancy and smoking cessation in early pregnancy

We performed the binary logistic regression analyses to estimate the risk of smoking while pregnant (Table 4). We did not find evidence for an association between the MFAS total score at the 24th and 34th gwks and smoking in pregnancy (aOR = 1.00–1.003, multiplicity adjusted *p* > 0.05) or the MFAS subscales and smoking in pregnancy (aOR = 0.97–1.04, multiplicity adjusted *p >* 0.05) while controlling for maternal age, education, single parenthood, parity, and concurrent symptoms of PPD.

**Table 4:** The results from the logistic regression models estimating the association between MFA and smoking at any time during pregnancy and the interaction effects MFAS × Education [low, middle, high] and MFAS × PPD on smoking at any time during pregnancy

	Smoking at any time during pregnancy						Smoking at any time during pregnancy						Smoking at any time during pregnancy					
	*β*	*S.E*	*p*	*adj. p*	*aOR*	*95% CI*	*β*	*S.E*	*p*	*adj. p*	*aOR*	*95% CI*	*β*	*S.E*	*p*	*adj. p*	*aOR*	*95% CI*
Intercept	-0.61	0.70	0.384		0.55	0.14-2.14	-0.73	0.91	0.424		0.48	0.08-2.88	-1.18	0.82	0.150		0.31	0.06-1.53
***MFAS 24 gwks***	0.00	0.01	0.937	1.000	1.00	0.99-1.01	0.00	0.01	0.832		1.00	0.99-1.02	0.01	0.01	0.364		1.01	0.99-1.02
*Education*																		
*Low*	Ref.						Ref						Ref.					
*Middle*	-1.23	0.15	<0.001**		0.29	0.22-0.39	-1.23	0.15	<0.001**		0.29	0.22-0.39	1.36	1.16	0.242		3.89	0.40-37.84
*High*	-1.76	0.17	<0.001**		0.17	0.12-0.24	-1.76	0.17	<0.001**		0.17	0.12-0.24	-2.29	1.38	0.099		0.10	0.01-1.56
*EPDS*	0.05	0.02	0.004**		1.05	1.02-1.09	0.07	0.10	0.463		1.07	0.89-1.30	0.05	0.02	0.004**		1.05	1.02-1.09
*SCL-90 Anxiety*	0.00	0.02	0.854		1.00	0.97-1.03	0.00	0.02	0.860		1.00	0.97-1.03	0.00	0.02	0.854		1.00	0.97-1.03
*MFAS × EPDS*							0.00	0.00	0.834	1.000	1.00	1.00-1.00						
*MFAS × Education low*													Ref.					
*MFAS × Education middle*													-0.03	0.01	0.026*	0.137	0.97	0.95-0.996
MFAS × *Education high*													0.01	0.02	0.698	1.000	1.01	0.98-1.04
Intercept	-0.83	0.72	0.251		0.44	0.11-1.79	-0.96	0.96	0.314		0.38	0.06-2.50	-1.25	0.85	0.138		0.29	0.05-1.50
***MFAS 34 gwks***	0.00	0.01	0.595	1.000	1.00	0.99-1.02	0.01	0.01	0.610		1.01	0.99-1.02	0.01	0.01	0.312		1.01	0.99-1.02
* Education*																		
*Low*	Ref.						Ref.						Ref.					
*Middle*	-1.26	0.15	<0.001**		0.28	0.21-0.38	-1.27	0.15	<0.001**		0.28	0.21-0.38	1.22	1.29	0.345		3.38	0.27-42.51
*High*	-1.76	0.17	<0.001**		0.17	0.12-0.24	-1.76	0.17	<0.001**		0.17	0.12-0.24	-2.70	1.54	0.080		0.07	0.00-1.38
* EPDS*	0.05	0.02	0.002**		1.06	1.02-1.09	0.08	0.11	0.476		1.08	0.87-1.34	0.05	0.02	0.002**		1.06	1.02-1.09
* SCL-90 Anxiety*	-0.02	0.02	0.191		0.98	0.94-1.01	-0.02	0.02	0.192		0.98	0.95-1.01	-0.02	0.02	0.209		0.98	0.95-1.01
* MFAS × EPDS*							0.00	0.00	0.832	1.000	1.00	1.00-1.00						
* MFAS × Education low*													Ref.					
* MFAS × Education middle*													-0.03	0.01	0.054	0.284	0.97	0.95.1.00
MFAS *×Education high*													0.01	0.02	0.538	1.000	1.01	0.98-1.04

The estimates are based on the multiple imputed data. At 24th gestational weeks, *N* = 2858. At 34th gestational weeks, *N* = 2858

The binary logistic regression models are controlled for maternal age, education, single parenthood, parity and concurrent depressive and anxiety symptoms

*S.E* Standard error, *adj. pP* value adjusted for multiple comparisons, *aOR* Adjusted odds ratio, *CI* Confidence interval, *MFAS* Maternal-Fetal Attachment Scale*, EPDS* Edinburgh (Pre) Postnatal Depression Scale, *SCL-90 Anxiety* Symptom Checklist -90 Anxiety Subscale

* *p*-value <.05, **p-value <.01

In the sample of pregnant smokers, we performed the binary logistic regression analyses to evaluate the relationship between MFA and cessation of smoking during pregnancy (Table 5). We did not observe evidence for the association between MFA (the total sum scores of MFAS at the 24th and 34th gwks) and quitting smoking during pregnancy (aOR = 1.02–1.02, multiplicity adjusted *p >* 0.05). However, a marginally significant association between the MFAS “*Giving of self”* subscale and smoking cessation in early pregnancy was demonstrated at 24 gwks (β = 0.13, aOR = 1.13, 95% CI [1.04, 1.24], *p* = 0.007, multiplicity adjusted *p* = 0.062). A one unit higher score in the MFAS subscale “*Giving of self”* at 34 gwks associated with an increased probability of smoking cessation (β = 0.16, aOR = 1.17, 95% CI [1.07, 1.29], *p* < 0.001, multiplicity adjusted *p* = 0.008). There was no evidence of associations between the other subscales of MFAS and smoking cessation during pregnancy (aOR = 0.99–1.05, multiplicity adjusted *p* > 0.05).

**Table 5:** The results from the logistic regression models estimating the association between MFA and smoking cessation in early pregnancy and the interaction effect MFAS × Education [low, middle, high] on cessation of smoking in early pregnancy

	Smoking cessation in early pregnancy						Smoking cessation in early pregnancy					
	*β*	*S.E*	*p*	*adj. p*	*aOR*	* 95% CI*	*β*	*S.E*	*p*	*adj. p*	*aOR*	*95% CI*
Intercept	-1.29	1.33	0.335		0.28	0.02-3.80	-1.22	1.49	0.412		0.29	0.02-5.51
***MFAS 24 gwks***	0.02	0.01	0.088	0.781	1.02	1.00-1.04	0.02	0.01	0.162		1.02	0.99-1.05
* Education*												
*Low*	Ref.						Ref.					
*Middle*	0.90	0.34	0.008**		2.45	1.26-4.76	0.48	2.46	0.844		1.62	0.01-204.11
*High*	1.17	0.43	0.007**		3.23	1.38-7.52	1.42	3.51	0.685		4.15	0.00-4144.87
* EPDS*	-0.03	0.03	0.451		0.98	0.91-1.04	-0.03	0.03	0.450		0.98	0.91-1.04
* SCL-90 Anxiety*	-0.02	0.03	0.621		0.99	0.93-1.04	-0.01	0.03	0.629		0.99	0.93-1.05
* MFAS × Education low*							Ref.					
* MFAS × Education middle*							0.01	0.03	0.865	1.000	1.01	0.95-1.06
MFAS *×Education high*							0.00	0.04	0.943	1.000	1.00	0.92-1.08
Intercept	-1.04	1.33	0.435		0.35	0.03-4.85	-0.62	1.48	0.676		0.54	0.03-9.86
***MFAS 34 gwks***	0.02	0.01	0.155	1.000	1.02	0.99-1.04	0.01	0.01	0.369		1.01	0.99-1.04
* Education*												
*Low*	Ref.						Ref.					
*Middle*	0.79	0.34	0.021*		2.20	1.12-4.30	-0.87	2.75	0.751		0.42	0.00-92.87
*High*	1.15	0.43	0.008**		3.15	1.35-7.33	-0.41	3.95	0.917		0.66	0.00-1569.18
* EPDS*	-0.04	0.03	0.179		0.96	0.90-1.02	-0.04	0.03	0.178		0.96	0.90-1.02
* SCL-90 Anxiety*	0.00	0.03	0.965		1.00	0.94-1.06	0.00	0.03	0.976		1.00	0.94-1.06
* MFAS × Education low*							Ref.					
* MFAS × Education middle*							0.02	0.03	0.545	1.000	1.02	0.96-1.08
MFAS *× Education high*							0.02	0.04	0.693	1.000	1.02	0.93-1.11

The estimates are based on the multiple imputed data. At 24th gestational weeks, *N* = 429. At 34th gestational weeks, *N* = 426

The binary logistic regression models are controlled for maternal age, education, single parenthood, parity and concurrent depressive and anxiety symptoms

*S.E* Standard error, *adj. pP* value adjusted for multiple comparisons, *aOR* Adjusted odds ratio, *CI* Confidence interval, *MFAS* Maternal-Fetal Attachment Scale*, EPDS* Edinburgh (Pre) Postnatal Depression Scale, *SCL-90 Anxiety* Symptom Checklist -90 Anxiety Subscale

* *p*-value <.05, **p-value <.01

### Maternal-fetal attachment modifying the effects of maternal education and prenatal psychological distress on smoking and smoking cessation during pregnancy

A lower level of maternal education and having more symptoms of prenatal depression associated with a higher risk of smoking in pregnancy. The participants who suffered from a higher level of depressive symptoms were more likely to smoke during pregnancy (aOR = 1.05–1.06, *p* = 0.002–0.004). Further, the probability of smoking in pregnancy was considerably lower among the women with a middle level (aOR = 0.28–0.29, *p* < 0.001) or high level of education (aOR = 0.17, *p* < 0.001) when compared with the probability of prenatal smoking among the women with the lowest educational level. The association between anxiety symptoms and smoking in pregnancy was not observed. Therefore, the interaction effects *MFAS* **×** Education [low, middle, high] and *MFAS* **×** PPD [EPDS] on smoking in pregnancy were estimated (Table 4). Our preliminary findings suggested that a higher level of MFA associated with a decrease in the risk of smoking among the women with a middle level of education when compared to the risk of smoking among the women with low (β = -0.03, aOR = 0.97, 95% CI [0.95, 0.996], *p* = 0.026, multiplicity adjusted *p* = 0.137) or high education (β = -0.04, aOR = 0.96, 95% CI [0.93, 0.998], *p* = 0.038, multiplicity adjusted *p* = 0.199). The effect of maternal education on smoking in pregnancy did not exhibit any difference in the way it was dependent on MFA among the women with a high education compared to the women with a low education (β = 0.006, aOR = 1.006, 95% CI [0.98, 1.04], *p* = 0.698, multiplicity adjusted *p* = 1.00). However, the evidence for the modifying effect of MFA on the association between maternal education and smoking in pregnancy was not considered sufficiently convincing because the results did not survive the corrections for multiple comparisons (multiplicity adjusted *p* > 0.05). Further, the modifying effect of MFA on the strength or direction of the association between prenatal depressive symptoms and smoking in pregnancy was not demonstrated.

In the population of pregnant smokers, the probability of smoking cessation during pregnancy was higher among the women with a middle level of education (aOR = 2.20–2.45, *p* = 0.008–0.021) and the women with a high level of education (aOR = 3.15–3.23, *p* = 0.007–0.008) when compared with the probability of smoking cessation among the women with the lowest educational level. However, we did not find evidence of an association between PPD (neither EPDS nor SCL-90 Anxiety subscale) and smoking cessation during pregnancy (aORs = 0.96–1.00, *p* = 0.179–0.965). Therefore, we estimated only the interaction effect of *MFAS* **×** Education [low, middle, high] on smoking cessation (Table 5). We did not confirm that the relationship between maternal education and smoking cessation during pregnancy was modified depending upon the level of MFA.

## Discussion

The aim of this study was to assess the role of MFA in prenatal smoking habits in a large population-based pregnancy cohort study with over 3700 participants. In this study, we did not find evidence of an association between the total score of MFA and smoking at any time during pregnancy or smoking cessation in early pregnancy. Nevertheless, a higher level of the altruistic dimension of MFA, *Giving of self*, associated with an increased probability of smoking cessation in early pregnancy. The second question in this study sought to determine the modifying role of MFA on the expected effects of maternal education and PPD on prenatal smoking behavior. The evidence for the modifying effect of MFA on the association between maternal education and smoking at any time during pregnancy was not considered sufficiently convincing as the result did not survive the correction for multiple comparisons. Further, we did not confirm that the association between maternal education and smoking cessation in early pregnancy was modified depending upon the level of MFA. The modifying effect of MFA on the strength or direction of the association between prenatal depressive symptoms and smoking in pregnancy was not demonstrated.

Our findings did not provide evidence for the association between MFA and maternal prenatal smoking behavior, and therefore MFA seems not to play such a significant role in the determination of smoking in pregnancy. This finding is contrary to previous studies suggesting that MFA significantly associates with positive maternal prenatal health practices [19, 20, 22], also with building healthier smoking habits [22, 23, 25]. Nevertheless, our most important finding suggested a one unit stronger altruistic attachment, that is a one point higher score in the *Giving of self* subscale of MFAS, associated with a 1.13–1.17 fold increase in the odds of smoking cessation in early pregnancy. Qualitative studies have revealed that the expectant mother’s willingness to do her best for the unborn child facilitates smoking cessation in pregnancy [40]. In addition, altruistic caregiving has been found to be the most significant life experience promoting abstinence in the context of addictions [41]. In the recent comprehensive review and meta-analysis maternal-fetal attachment was not yet recognized as a predictor of smoking cessation during pregnancy [16]. Our finding addressing the beneficial association between maternal altruistic attachment towards the fetus and cessation of smoking during pregnancy replicates the previous finding of Massey et al. (2015) and contributes to prior knowledge by providing empirical evidence that especially maternal desire and endeavor to help the fetus offers a motivational stance for quitting smoking in pregnancy.

The total level of MFA was equal among the non-smokers and the smokers, but the women who smoked scored higher in some of the subscales of MFAS. Our study revealed that of the women who smoked those who were able to quit smoking scored higher in the one subscale of MFAS, “*Giving of self*”. Prior research has addressed the importance of this particular dimension of MFA as a predictor of smoking cessation [25]. Interestingly, pregnant smokers considering smoking cessation have been shown to display a stronger attachment towards the fetus than pregnant women who have never smoked [24]. This finding has been explained by cognitive dissonance caused by an awareness of the harmful effects of smoking on the fetus and the awakening of prenatal attachment; this results in a tendency to resolve the contradicting situation by preparing to quit smoking, which may affirm attachment [24].

Our descriptive sample characteristics revealed that the smoking women, and particularly the persistent smokers, constitute high-risk populations among pregnant women as regards the level of PPD, the prevalence of prenatal depression and maternal educational attainment. Our study confirmed the adverse associations of low maternal education and prenatal depressive symptoms with smoking in pregnancy, which is consistent with the previous literature [5, 7, 8, 28, 34]. The finding to emerge from our analyses is that other underlying mechanisms, for example those related to maternal educational disadvantage and poor maternal prenatal mental health as regards symptoms of depression, seem to have a stronger relationship with prenatal smoking habits than MFA. In contrast to earlier findings [16, 42], the adverse association between prenatal anxiety and smoking habits during pregnancy was not substantiated in the current study. Further, no evidence for the detrimental associations between PPD and smoking cessation in early pregnancy was detected in the current study, which is contrary to previous findings [16]. However, the lower academic achievements of the expectant mothers associated with a lower probability of smoking cessation in early pregnancy which accords with previous studies [7, 16]. To our knowledge, prenatal parenting – MFA – has not previously been evaluated as a potential moderator of the effects of maternal education and PPD on smoking behavior in pregnancy. The current study found that the level of MFA was the highest among the women with the lowest educational attainment. Previous studies investigating the level of MFA in relation to maternal education have reported inconclusive findings [43, 44]. Our preliminary finding suggested that stronger MFA associated with a decrease in the risk of smoking in pregnancy among the women with a middle level of education when compared to the risk of smoking among the pregnant women with a low or high education level. However, the evidence for the modifying effect of MFA on the association maternal education and smoking in pregnancy was not considered convincing as the result did not survive the correction for multiple comparisons. Further, we did not confirm the modifying effect of MFA on the strength or direction of the association between prenatal depressive symptoms and smoking in pregnancy or the association between maternal education and cessation of smoking in early pregnancy. Therefore, the potentially protective moderation effect of MFA was not demonstrated in the presence of educational disadvantage or prenatal depressive symptoms, both of which presented with independent associations with smoking behavior during pregnancy. These negative findings may refer to the fact that MFA is not sufficiently efficient to modify the associations between maternal education/PPD and maternal smoking behavior in a protective manner. For several reasons, it is necessary to interpret the result carefully. First, our variable describing the participants’ educational attainment was relatively rudimentary. In addition, a cross-sectional study is not able to consider longitudinal interferences. Smoking in pregnancy has been perceived as an unfavorable cascade starting from the mother’s childhood or adolescence [45–48] and strongly predicted by socioeconomic factors and environmental influences [7, 9, 46, 47, 49], which precede an awakening of MFA. On the other hand, the transition to parenthood may already inspire a change in smoking habits in the preconception period; many smokers, especially women with higher education, quit smoking just before pregnancy [50, 51].

The FinnBrain Birth Cohort has shown to be well representative of the source population [52]. The survey data suggested a somewhat lower prevalence of prenatal smoking than in the general population of the same hospital district (12.7% vs. 16.6%, respectively) [52]. Therefore, the register data was linked with the survey self-reports leading to an approximately similar prevalence of smoking in pregnancy than presented in the general population of the same area [52] and in the Finnish national statistics [53]. Unfortunately, smoking in pregnancy is still found to be considerably more common in Finland than in the other Scandinavian countries [5] and in many European regions [4]. The present study showed a cessation rate of 58%, which is slightly higher than the cessation rate of 39–49% presented in the Finnish national statistics [53] and a frequent cessation rate of less than 50% reported in the previous population based studies [4]. A relatively high level of maternal education and attendance in prenatal care may have contributed to the high cessation rate [16].

### Strengths and limitations

A large sample size and a study design integrating the data from a prospective birth cohort survey with good quality register data can be considered the important strengths in the present study. However, several limitations should be noted. Due to the cross-sectional design a conclusion regarding causality cannot be drawn.

Our results, mainly negative, may also be partly related to limitations in the methodology. Despite the many years of research, there is still a lack of consensus about the construct of MFA and its assessment [54]. In this study, MFA was evaluated with a self-report measure, which primarily delineates behavioral aspects of prenatal attachment [26, 55]. Consequently, we may not have been able to capture the phenomenon of MFA completely [54–56], and the question of how critical a role the awakening of MFA plays in prenatal smoking behavior remains partly open. In addition, the subscales of MFAS have been criticized [55, 57, 58]. In the present study, the internal consistencies of some of the MFAS subscales were unfortunately found to be relatively weak which may, to some extent, undermine our results.

In addition, we may not have been able to control for all the confounding factors. The mother’s altruistic personality traits have shown to promote smoking cessation during pregnancy [59], but maternal personality characteristics were not controlled for in this study. Addictiveness of tobacco smoking and the severity of nicotine dependence may override the health promoting potential of MFA as tobacco products are considered to exert the strongest dependence potential among addictive substances [60]. Heavier smoking and more severe nicotine dependence have shown to predict a lower rate of smoking cessation during pregnancy [16, 25]. Prenatal smoking behavior was not biochemically verified, and the severity of nicotine dependence and possible interventions for tobacco use disorder were not adjusted for. In addition, the relationship between MFA and prenatal smoking habits may be multidirectional and complex. Addictions have been shown to diminish the reward from caregiving and the regulation of stress, as the neural circuitries essential in both overlap with the neuronal pathways dysregulated in substance use disorders [61]. This may also apply to maternal processes implicated in sensitive parenting and tobacco use disorder. The same conditions, such as adverse childhood experiences and lack of social support, may influence both MFA and smoking in pregnancy [62–64].

## Conclusions

Psychosocial interventions have shown to increase the smoking cessation rate in pregnancy [65]. Nevertheless, only 13% of pregnant smokers reach and maintain abstinence after receiving a smoking cessation intervention [66]. Qualitative studies have addressed the fact that pregnant women who smoke rarely consider smoking cessation interventions to enable them to quit whereas the desire to be a good mother, to protect the fetus, and to quit smoking for the baby’s sake are perceived as facilitating smoking cessation [40]. Our study shows that the stronger level of altruistic dimension of maternal-fetal attachment associates with an increased probability of smoking cessation and suggests it should be given more emphasis in prenatal smoking cessation interventions. It is noteworthy that several interventions to enhance maternal-fetal attachment have already been introduced [29]. The aim of these interventions is to enhance the mother’s emotional wellbeing, her awareness of the fetus and her abilities to conceptualize the unborn child as an individual person, as well as providing antenatal education, social and psychological support [29]. Maternal-fetal attachment including the altruistic caregiving relationship with the unborn child can potentially be strengthened [29] which may constitute a promising novel approach for interventions aiming at promoting cessation of smoking and other substance use during pregnancy [67].

## Data Availability

The dataset that support the findings of this study has been constructed by combining survey data from The FinnBrain Birth Cohort Study and register data from The Finnish Medical Birth Register. Restrictions apply to the availability of data obtained from The Finnish Medical Birth Register and thus, the datasets generated and analyzed during the current study are not publicly available due to an obligation to maintain confidentiality of The Finnish Medical Birth Register records.

## Abbreviations

MFA: Maternal-fetal attachment
PPD: Prenatal psychological distress
gwks: Gestational weeks
MFAS: Maternal-Fetal Attachment Scale
EPDS: Edinburgh Pre/Postnatal Depression Scale
SCL-90: Symptom Checklist-90
