# Correction to: The association of maternal-fetal attachment with smoking and smoking cessation during pregnancy in The FinnBrain Birth Cohort Study

**DOI:** 10.1186/s12884-021-03771-z

**Published:** 2021-04-10

**Authors:** Heidi Jussila, Juho Pelto, Riikka Korja, Eeva Ekholm, Marjukka Pajulo, Linnea Karlsson, Hasse Karlsson

**Affiliations:** 1 Department of Child Psychiatry, Doctoral Programme of Clinical Investigation, University of Turku, Turku, Finland; 2 Department of Clinical Medicine, FinnBrain Birth Cohort Study, Turku Brain and Mind Center, University of Turku, Lemminkäisenkatu 3, 20014 Turku, Finland; 3 Department of Psychology, University of Turku, Turku, Finland; 4 Department of Obstetrics and Gynecology, Turku University Hospital, Turku, Finland; 5 Department of Child Psychiatry, University of Turku, Turku, Finland; 6 Centre for Population Health Research, University of Turku and Turku University Hospital, Turku, Finland; 7 Department of Child Psychiatry, Turku University Hospital and University of Turku, Turku, Finland; 8 Department of Psychiatry, Turku University Hospital and University of Turku, Turku, Finland

**Correction to: BMC Pregnancy Childbirth 20, 741 (2020)**

**https://doi.org/10.1186/s12884-020-03393-x**

Following publication of the original article [1], the authors reported an error in Tables 2 and 3 headings of the two columns are missing during layout processing. The correct tables are given below.

**Table 2:** The descriptives statistics of MFA and PPD, and the comparisons between the pregnant smokers and the non-smokers

	Non-smokers			Smokers			Between-groups difference	
	*N* = 3086			*N* = 612				
	*n*	*Mean (SD)*	*Median (range)*	*n*	*Mean (SD)*	*Median (range)*	*p*^*a*^	*r*
**24gwks**								
MFAS	2331	86.9 (10.6)	87 (51–116)	409	88.0 (10.0)	88 (44–113)	0.074	0.03
*Role taking*	2336	16.7 (2.8)	17 (6–20)	410	16.8 (2.7)	17 (7–20)	0.703	0.01
*Differentiation*	2340	15.4 (2.5)	15 (6–20)	410	15.7 (2.4)	16 (7–20)	0.032*	0.04
*Interacting*	2338	16.6 (3.1)	17 (5–25)	410	17.1 (3.0)	17 (8–24)	0.004**	0.05
*Attributing*	2335	18.5 (3.4)	19 (6–30)	409	19.0 (3.2)	19 (11–29)	0.006**	0.05
*Giving*	2338	19.7 (2.5)	20 (9–25)	410	19.5 (2.5)	20 (8–25)	0.091	0.03
EPDS	2317	4.8 (4.1)	4 (0–25)	408	6.0 (4.3)	5 (0–23)	< 0.001**	0.11
SCL-90 Anxiety	2314	3.8 (4.2)	3 (0–30)	408	4.7 (4.6)	4 (0–24)	< 0.001**	0.08
**34gwks**								
MFAS	2188	92.0 (10.2)	93 (52–116)	373	93.1 (10.2)	93 (55–117)	0.137	0.03
*Role taking*	2202	17.1 (2.6)	18 (7–20)	374	17.1 (2.5)	18 (8–20)	0.886	0.00
*Differentiation*	2204	15.9 (2.4)	16 (8–20)	374	16.2 (2.2)	16 (10–20)	0.111	0.03
*Interacting*	2202	18.1 (3.1)	18 (5–25)	374	18.5 (2.8)	19 (10–25)	0.037*	0.04
*Attributing*	2198	21.1 (3.4)	22 (9–30)	373	21.8 (3.4)	22 (12–30)	0.001**	0.07
*Giving*	2198	19.8 (2.4)	20 (10–25)	374	19.6 (2.5)	20 (10–25)	0.171	0.03
EPDS	2191	4.7 (4.0)	4 (0–26)	373	5.9 (4.5)	5 (0–23)	< 0.001**	0.10
SCL-90 Anxiety	2186	3.1 (3.8)	2 (0–27)	374	3.8 (4.6)	3 (0–33)	0.012*	0.05
		**n/N**	**%**		**n/N**	**%**	***Χ***^***2***^ ***p***	**Cramer’s ϕ**
**24 gwks** EPDS≥10		299/2317	12.9%		73/408	17.9%	0.007**	0.05
**34 gwks** EPDS≥10		287/2191	13.1%		84/373	22.5%	< 0.001**	0.09

The estimates are based on the original data, not the imputed dataset

Attrition in the MFAS questionnaire responses was higher among the smoking women in comparison with the non-smokers (24 gwks: 33.2% vs. 24.5%, χ2 = 20.2, *p* < 0.001; 34 gwks: 39.1% vs. 29.1%, χ2 = 23.8, *p* < 0.001, respectively)

*MFAS* Maternal-Fetal Attachment Scale and the subscales of Maternal-Fetal Attachment Scale: *Role taking*, *Differentiation*, Differentiation of self from fetus, *Interacting*, Interacting with the fetus, *Attributing*, Attributing characteristics to the fetus and *Giving* Giving of self, *EPDS* Edinburgh (Pre) Postnatal Depression Scale, *SCL-90 Anxiety* Symptom Checklist − 90, the Anxiety Subscale

* *p*-value <.05, **p-value <.01

^a^ Mann-Whitney U test

**Table 3:** The descriptives statistics of MFA and PPD, and the comparisons between the smokers who quit and the persistent smokers

	Quit smoking			Continued smoking			Between-groups difference	
	*N* = 354			*N* = 255				
	*n*	*Mean (SD)*	*Median (range)*	*n*	*Mean (SD)*	*Median (range)*	*p*^*a*^	*r*
**24 gwks**								
MFAS	257	88.6 (10.0)	88 (57–113)	152	87.0 (10.0)	87 (44–112)	0.102	0.08
*Role taking*	258	16.6 (2.7)	17 (8–20)	152	16.7 (2.6)	17 (7–20)	0.237	0.06
*Differentiation*	258	15.7 (2.4)	16 (9–20)	152	15.6 (2.3)	16 (7–20)	0.644	0.02
*Interacting*	258	17.1 (3.1)	17 (8–24)	152	17.0 (2.9)	17 (9–24)	0.574	0.03
*Attributing*	257	19.1 (3.2)	19 (11–29)	152	19.0 (3.2)	19.0 (11–29)	0.773	0.01
*Giving*	258	19.8 (2.4)	20 (13–25)	152	18.8 (2.6)	19 (8–25)	< 0.001**	0.18
EPDS	256	5.6 (4.1)	5 (0–20)	152	6.8 (4.5)	6 (0–23)	0.006**	0.14
SCL-90 Anxiety	256	4.3 (4.5)	3 (0–24)	152	5.4 (4.7)	4 (0–20)	0.007**	0.13
**34 gwks**								
MFAS	236	93.9 (9.7)	93 (67–115)	137	91.8 (10.9)	91 (55–117)	0.093	0.09
*Role taking*	236	17.3 (2.5)	18 (8–20)	138	17.0 (2.5)	17 (8–20)	0.136	0.08
*Differentiation*	236	16.2 (2.3)	16 (10–20)	138	16.1 (2.2)	16 (10–20)	0.883	0.01
*Interacting*	236	18.5 (2.7)	19 (12–25)	138	18.4 (3.0)	18 (10–25)	0.555	0.03
*Attributing*	236	21.9 (3.3)	22 (12–28)	137	21.6 (3.7)	22 (12–30)	0.542	0.03
*Giving*	236	20.1 (2.5)	20 (12–25)	138	18.8 (2.5)	18 (10–25)	< 0.001**	0.26
EPDS	235	5.4 (4.2)	5 (0–20)	138	6.8 (4.9)	6 (0–23)	0.007**	0.14
SCL-90 Anxiety	235	3.5 (4.5)	2 (0–33)	139	4.5 (4.7)	3 (0–26)	0.016*	0.12
		**n/N**	**%**		**n/N**	**%**	***Χ***^***2***^***p***	**Cramer’s ϕ**
**24 gwks** EPDS≥10		36/256	14.1%		37/152	24.3%	0.009**	0.13
**34 gwks** EPDS≥10		42/235	17.9%		42/138	30.4%	0.005**	0.15

The estimates are based on the original data, not the imputed dataset

Attrition in the MFAS questionnaire responses was higher among the persistent smokers in comparison with the women who quit smoking during pregnancy (24gwks: 40.9% vs. 26.0, χ2 = 14.5, *p* < 0.001; 34 gwks: 46.8% vs. 29.6%, χ2 = 18.2, *p* < 0.001, respectively)

*MFAS* Maternal-Fetal Attachment Scale and the subscales of Maternal-Fetal Attachment Scale: *Role taking*, *Differentiation* Differentiation of self from fetus, *Interacting* Interacting with the fetus, *Attributing* Attributing characteristics to the fetus and *Giving* Giving of self*, EPDS* Edinburgh (Pre) Postnatal Depression Scale, *SCL-90 Anxiety* Symptom Checklist − 90, the Anxiety Subscale

* *p*-value <.05, **p-value <.01

^a^ Mann-Whitney U test
